# Endocannabinoid and Cannabinoid-Like Fatty Acid Amide Levels Correlate with Pain-Related Symptoms in Patients with IBS-D and IBS-C: A Pilot Study

**DOI:** 10.1371/journal.pone.0085073

**Published:** 2013-12-27

**Authors:** Jakub Fichna, JodiAnne T. Wood, Malvina Papanastasiou, Subramanian K. Vadivel, Piotr Oprocha, Maciej Sałaga, Marta Sobczak, Anna Mokrowiecka, Adam I. Cygankiewicz, Piotr K. Zakrzewski, Ewa Małecka-Panas, Wanda M. Krajewska, Piotr Kościelniak, Alexandros Makriyannis, Martin A. Storr

**Affiliations:** 1 Snyder Institute for Chronic Diseases, University of Calgary, Calgary, Alberta, Canada; 2 Department of Medicine, Division of Gastroenterology, University of Calgary, Calgary, Alberta, Canada; 3 Department of Biomolecular Chemistry, Faculty of Medicine, Medical University of Lodz, Lodz, Poland; 4 Department of Digestive Tract Diseases, Faculty of Medicine, Medical University of Lodz, Lodz, Poland; 5 Center for Drug Discovery, Northeastern University, Boston, Massachusetts, United States of America; 6 Faculty of Applied Mathematics, AGH University of Science and Technology, Cracow, Poland; 7 Department of Cytobiochemistry, Faculty of Biology and Environmental Protection, University of Lodz, Lodz, Poland; 8 Faculty of Mathematics and Computer Science, Jagiellonian University, Cracow, Poland; 9 Department of Medicine, Division of Gastroenterology, Ludwig Maximilians University of Munich, Munich, Germany; Medical University of Gdańsk, Poland

## Abstract

**Aims:**

Irritable bowel syndrome (IBS) is a functional gastrointestinal (GI) disorder, associated with alterations of bowel function, abdominal pain and other symptoms related to the GI tract. Recently the endogenous cannabinoid system (ECS) was shown to be involved in the physiological and pathophysiological control of the GI function. The aim of this pilot study was to investigate whether IBS defining symptoms correlate with changes in endocannabinoids or cannabinoid like fatty acid levels in IBS patients.

**Methods:**

AEA, 2-AG, OEA and PEA plasma levels were determined in diarrhoea-predominant (IBS-D) and constipation-predominant (IBS-C) patients and were compared to healthy subjects, following the establishment of correlations between biolipid contents and disease symptoms. FAAH mRNA levels were evaluated in colonic biopsies from IBS-D and IBS-C patients and matched controls.

**Results:**

Patients with IBS-D had higher levels of 2AG and lower levels of OEA and PEA. In contrast, patients with IBS-C had higher levels of OEA. Multivariate analysis found that lower PEA levels are associated with cramping abdominal pain. FAAH mRNA levels were lower in patients with IBS-C.

**Conclusion:**

IBS subtypes and their symptoms show distinct alterations of endocannabinoid and endocannabinoid-like fatty acid levels. These changes may partially result from reduced FAAH expression. The here reported changes support the notion that the ECS is involved in the pathophysiology of IBS and the development of IBS symptoms.

## Introduction

 Irritable bowel syndrome (IBS) is a functional gastrointestinal (GI) disorder, associated with alterations of bowel function and other symptoms related to the GI tract, such as abdominal pain or cramping (localized, intermittent pain), bloating or feeling of incomplete evacuation. The prevalence of IBS is estimated at up to 20% in the Western countries [1,2] and accounts for more than 50% of referrals to GI specialists [3–5]. IBS has a major impact on patient’s quality of life, and accounts for a heavy burden for, patients, health care providers and economies. Due to the lack of efficient treatments, there is an unmet need to develop novel anti-IBS strategies.

 The design of novel therapeutics to be used in IBS is impaired by our incomplete understanding of the pathophysiology of IBS and therefore the poor control of the changes in endogenous systems during IBS development. Therefore the systems that are well-known for their involvement in pain signalling and GI motility are currently regarded as being a main potential target for future anti-IBS drugs. The endogenous cannabinoid system (ECS), which consists of three principal pillars, i.e. “classical” cannabinoid (CB1, CB2) and “non-classical” (TRPV1, GPR55) receptors, endogenous ligands (anandamide, AEA; 2-arachidonyloglycerol, 2-AG) and the cluster of enzymes, responsible for CB synthesis and degradation [6] has recently attracted much attention as a crucial site in IBS pathophysiology. The localization of CB1 and CB2 receptors and their endogenous ligands in the GI tract and in sensory endings on primary sensory afferents [7–11], as well as participation of the ECS in pain signalling, modulation of sensory afferent information and GI peristalsis (for review, see: [6,12]) have been well established. However, much attention has currently been given to the fatty acid amide hydrolase (FAAH) and its metabolites. FAAH is an intracellular enzyme, primarily located in the liver and the brain, as well as in peripheral organs, like the GI tract (for review, see: [13]). It is commonly accepted that AEA is the main substrate for FAAH, but a growing body of evidence points that 2-AG, although not exclusively, is also deactivated by this hydrolase [14]. FAAH is also involved in the degradation of several other biolipids, including palmitoylethanolamide (PEA) and oleoylethanolamide (OEA), which may bind to both, “classical” and “non-classical” CB receptors. Therefore it has been suggested that the modulators of the FAAH activity, as potential anti-IBS therapeutics, may provide finer tuning of the ECS function than the CB receptor ligands, also in terms of peripheral vs. central site-dependent site effects.

 To further clarify whether the ECS is involved in IBS pathophysiology and whether changes in ECB or cannabinoid-like fatty acid amide levels contribute to clinical symptoms we established ECB and cannabinoid-like fatty acid amide levels in female patients with IBS. The levels where then correlated with IBS subtypes, namely IBS-D and IBS-C and, additionally, with the self-reported symptom scores and compared to age-matched female control group. Finally, FAAH mRNA levels were evaluated in colonic biopsies from female IBS-D and IBS-C patients and matched controls to investigate whether changes in FAAH expression at the transcription level contribute to the observed changes in metabolite concentrations. The correlations observed may provide a new understanding of the mechanisms underlying IBS and disturbances associated with GI functional and abdominal pain processing.

## Materials and Methods

### Ethics Statement

Human studies were approved by the Ethics Committee of the University of Calgary, Canada and Medical University of Lodz, Poland. All participating subjects gave written, informed consent prior to genetic analysis.

### Patients

Endocannabinoid and cannabinoid-like fatty acid amide levels were quantified in plasma from female IBS-D and IBS-C patients and healthy controls at the GI-clinic of the University of Calgary, Canada. In total 21 blood samples were analyzed. The study population comprised 7 patients with IBS-D (age 26-58), 7 patients with IBS-C (age 21-62), and 7 healthy subjects (age 29-56).

To quantify the relative expression of human FAAH, forceps biopsy samples were analyzed. In total 16 samples prepared from human colon biopsies were used for the study. The study population comprised 3 patients with IBS-D (age 43-71), 3 patients with IBS-C (age 25-62) and 10 healthy, unrelated controls (age 33-82) recruited from January 2011 to December 2012 at the Medical University of Lodz, Poland.

The diagnosis of IBS-D and IBS-C was assessed according to established clinical criteria using endoscopic, radiologic, and histopathologic examinations.

### Endocannabinoid and and cannabinoid-like fatty acid amide extraction from plasma

After collection, human plasma samples from each patient were kept at room temperature for 1,30, 60 and 120 minutes and were then immediately stored at -80°C until they were processed and analyzed. The extraction procedure for the standards, made in 20 mg/mL fatty acid free BSA, quality control samples, made in BSA, unextracted quality control samples, made in ethanol, and plasma samples was a modified version of the Folch extraction [15–17]. Proteins were precipitated in the standards, quality controls made in BSA and plasma samples using ice cold acetone:PBS, pH 7.4 (3:1) and internal standard, followed by centrifugation at 13,000 g for 5 minutes at 4°C. The resulting supernatant from all samples was dried under nitrogen until the acetone was removed. To the remaining supernatant, 100 µL PBS, one volume of methanol and two volumes of chloroform was added for liquid-liquid phase extraction of the lipids. The two phases were separated by centrifugation and the bottom organic layer was evaporated to dryness under nitrogen. Samples were reconstituted in 50 µL ethanol and were vortexed, sonicated briefly and centrifuged prior to analysis. 

### Nano LC-MS analysis for endocannabinoids

Samples were loaded on a C4 LC trap (5 µm, 300 A, Bruker-Michrom, Auburn, CA, USA) with water/acetonitrile/formic acid (80/20/0.1) at a flow rate of 10 µL/min for 3 min. Chromatographic separation was achieved using a Michro-Tech Scientific C18 column (50 mm, 75 µm ID, 3 µm particle, 120 A pore size) on an ABI 4000 Q-Trap mass spectrometer with a Tempo nano-LC on the front end (Applied Biosystems Inc., Framingham, MA). The mobile phase consisted of water/acetonitrile (95/5; A) and acetonitrile/water (95/5; B), with 0.1% formic acid in both, in the following two step gradient: initial conditions of 80% A decreased linearly to 20% A over 1 minute and to 100% B in 9 min. The system was held at these conditions for 5 minutes before returning to initial conditions (flow rate = 700 nl/min); the autosampler was kept at 4°C to prevent analyte degradation. The sample injection volume was 1µL and the reagent used to fill the loop (10 µL) was water/acetonitrile (95/5) with 0.1% formic acid. Eluted peaks were ionized using a nanoESI source on a 4000Qtrap (Applied Biosystems) in positive mode and detected by multiple reaction monitoring (MRM). Deuterated internal standards were used for each standard curve and the levels of each endocannabinoid or cannabinoid-like fatty acid amide per mL plasma were determined.

Standard curves were linear with a regression value of >0.993. Extraction efficiencies were greater than 85% for each compound and deuterated standard.

### Real-time reverse transcriptase (RT) PCR and quantification of FAAH expression in human biopsies

The biopsy specimens were immediately frozen after isolation and kept at -70°C until processing. The RNA was isolated using the PureLink RNA Mini kit (Life Technologies, Carlsbad, CA, USA) according to manufacturer’s protocol. The purity and quantity of isolated RNA was measured using dedicated spectrophotometer (BioPhotometer; Eppendorf, Germany). Total RNA (1 μg) was used for cDNA synthesis using a first strand cDNA synthesis kit (Fermentas, Canada). Quantitative analysis was performed using fluorescently labeled TaqMan probes Hs00155015_m1 and Hs01003267_m1 for human FAAH and hypoxanthine-guanine phosphoribosyltransferase (HPRT, endogenous control), respectively (Life Technologies, Carlsbad, CA, USA) on Mastercycler S realplex 4 apparatus (Eppendorf, Germany). All experiments were performed in triplicates.

The Ct values for studied genes were normalized to Ct values obtained for a housekeeping gene HPRT. Relative amount of mRNA copies was calculated using 2^-ΔCt method. For presentation, relative copy number values were recalculated to number of copies of each studied gene per 1000 copies of HPRT. The mean Ct values for the housekeeping gene (HPRT) in samples from all patient groups did not alter regardless of the studied group.

### Statistical analysis

Statistical analysis was performed in R software environment for statistical computing.

Level of significance was set in all tests to *P* ≤ 0.05. Statistical significance in the differences in levels (measured after 0, 30, 60 and 120 min) of AEA, 2-AG, OEA and PEA for different symptoms was tested using multivariate analysis of variance (MANOVA; 4 different tests were used: Pillai, Wilks, Hotelling-Lawley and Roy test). Each symptom was treated separately and compared with control group. In the case of symptoms, which showed difference in levels, an analysis of variance was applied (one-way ANOVA) to each measurement time (0, 30, 60, and 120 min). The LSD test was used in post hoc analysis (with Holm's p value adjustment). To establish correlations between disease type and specific symptoms, the χ^2^ test with Bonferroni correction was used.

 ANOVA followed by Bonferroni post-hoc testing was used to assess the differences among relative FAAH mRNA levels. *P* values < 0.05 were considered statistically significant.

 Figures were prepared using Prism 5.0 (GraphPad Software Inc., La Jolla, CA, USA).

## Results

### Endocannabinoid and cannabinoid-like fatty acid amide plasma levels change with stool pattern in IBS

**Figure 1:**
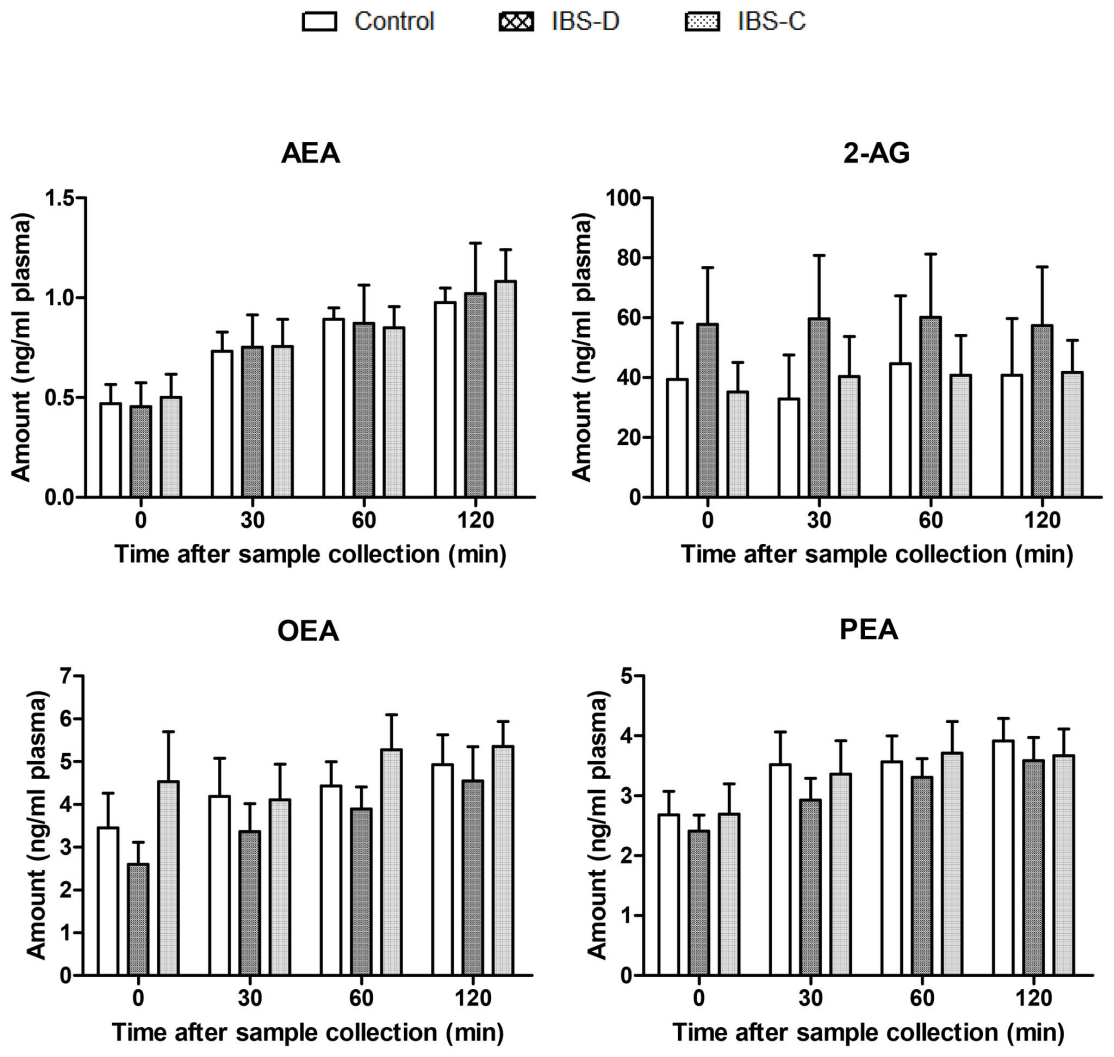
Endocannabinoid (anandamide, AEA; 2-arachidonoyl glycerol, 2-AG) and cannabinoid-like fatty acid amide (*N*-oleoyl ethanolamine, OEA; *N-*palmitoyl ethanolamine, PEA) levels in plasma 0, 30, 60 and 120 min after blood sample collection from IBS-D and IBS-C patients vs. **healthy controls**. The results are shown as mean ± SEM of n=7 for each group. Each sample was run in triplicate.

Endocannabinoid (AEA, 2-AG) and cannabinoid-like fatty acid amide (OEA, PEA) levels were detected in all plasma samples from IBS-D and IBS-C patients and healthy controls. In all patients AEA, OEA and PEA levels increased, while 2-AG level did not change over the time of the study, suggesting the release from peripheral blood cells, an observation which is congruent with earlier reported results [17]. In IBS-D patients, 2-AG levels were higher, whereas OEA and PEA levels were lower than in healthy controls (Figure 1). In contrast, OEA plasma levels were higher in IBS-C patients vs. healthy subjects.

MANOVA analysis showed no significant correlation between endocannabinoid and cannabinoid-like fatty acid amide plasma levels and IBS type.

### IBS type is significantly correlated with specific symptoms

 As expected, the abdominal pain was significantly more common in IBS-D (*p*= 0.0009995002 *vs.* controls) and IBS-C patients (*p*=0.001999 *vs.* controls) compared to healthy subjects. Also, there was a significant correlation between dysmotility symptoms and the IBS type (*p*=0.001999 for IBS-D and *p*=0.00149925 for IBS-C *vs.* controls).

Of note, IBS-D, but not IBS-C patients suffered from abdominal cramping (*p*=0.0009995002) in comparison with healthy controls.

### PEA plasma levels are correlated with abdominal cramping frequency in IBS patients

**Figure 2:**
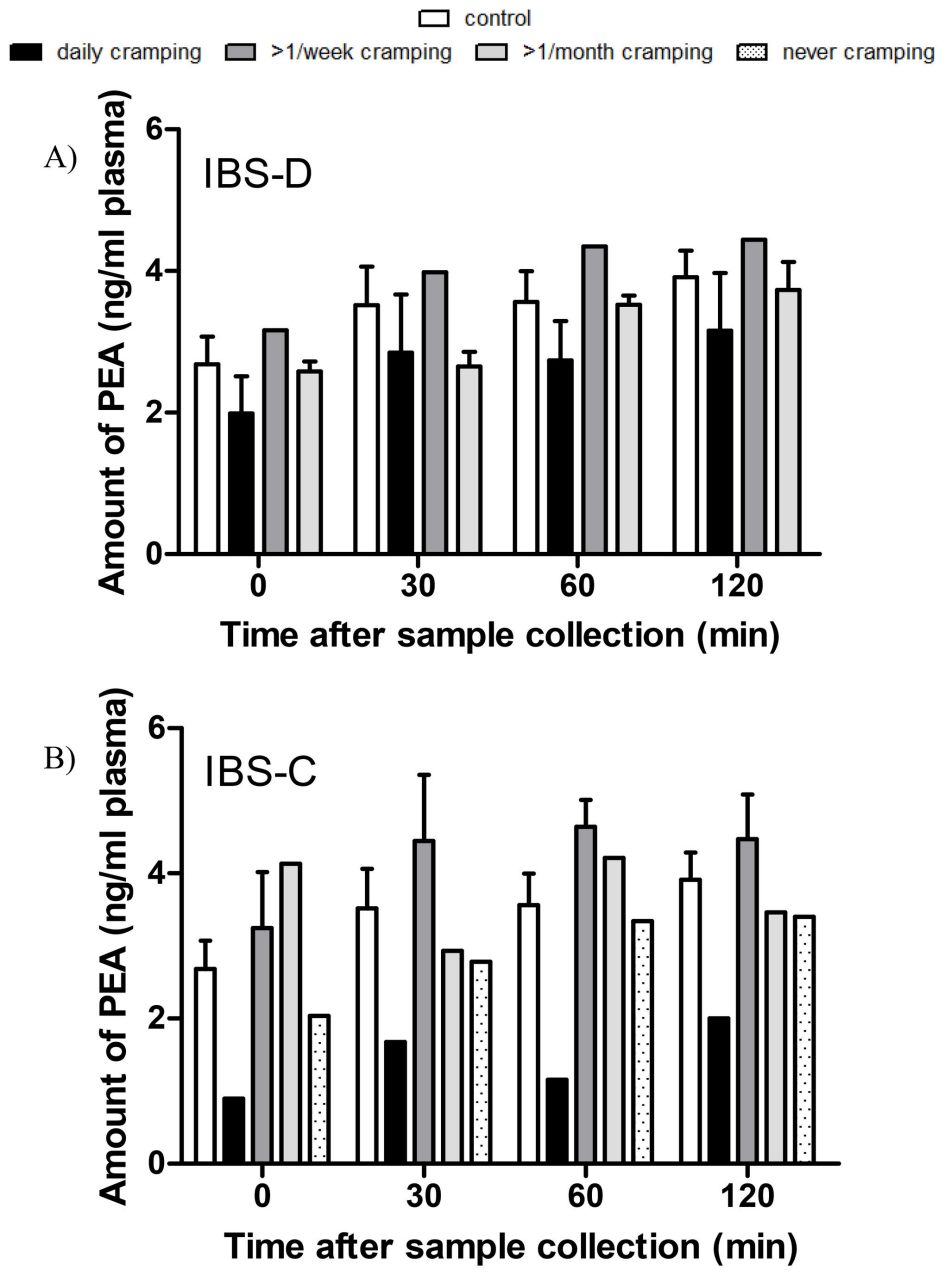
PEA levels in plasma from IBS patients. PEA levels in plasma 0, 30, 60 and 120 min after blood sample collection from IBS-D (A) and IBS-C (B) patients vs. abdominal cramping frequency. The results are shown as mean ± SEM of n=7 for each group. Each sample was run in triplicate.

 As shown in Figure 2, PEA levels were considerably lower in IBS-D and IBS-C patients with daily cramping, but equal or increased in patients suffering from >1/week or less frequent cramping, compared to healthy subjects. Low levels of PEA were detected in IBS-C patients with no symptoms of cramping (Figure 2).

### Decreased FAAH expression in IBS-C patients vs. healthy controls

**Figure 3:**
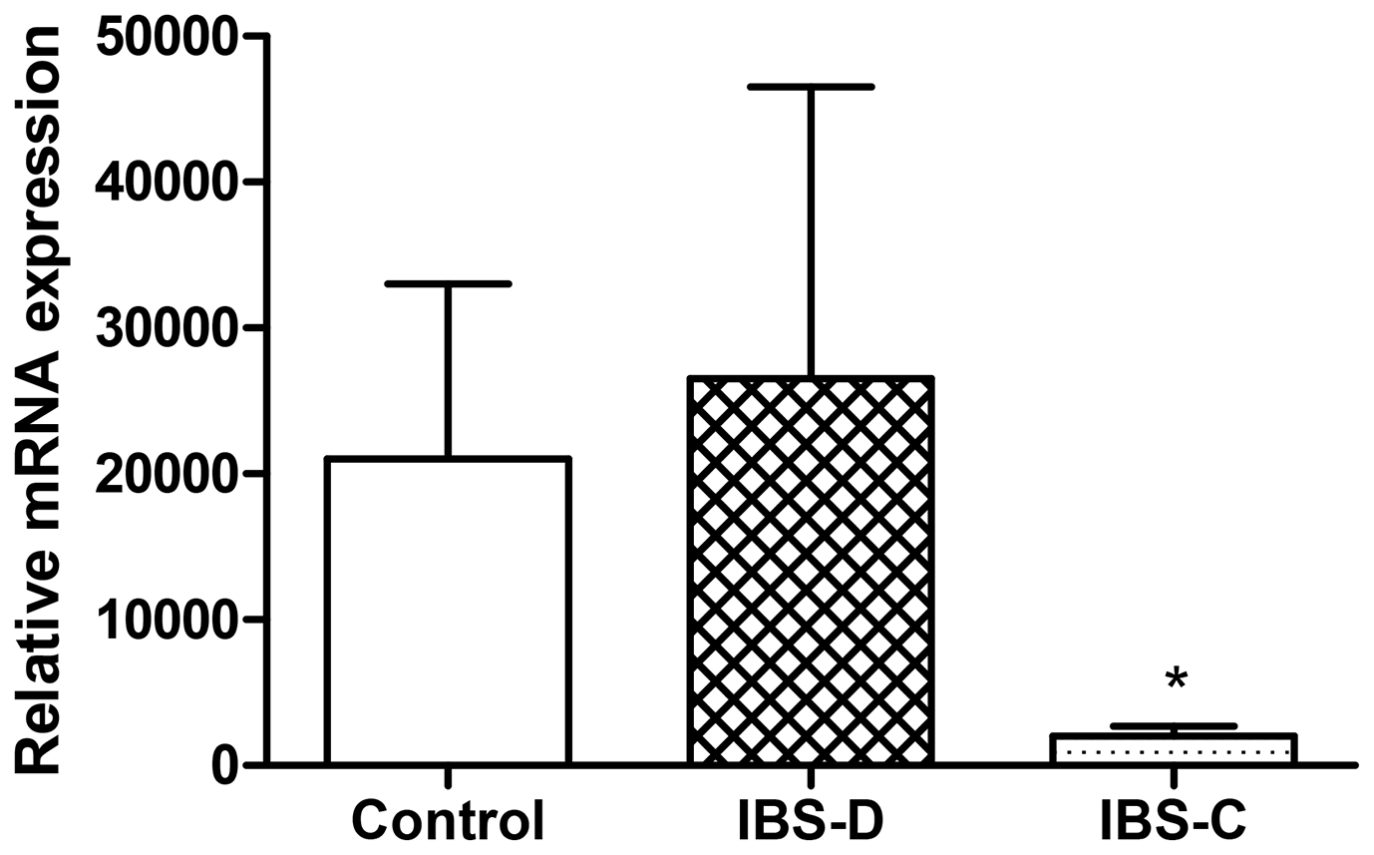
FAAH mRNA expression in colonic biopsies from patients with IBS-D and IBS-C *vs.* healthy controls. The results are shown as mean ± SEM.

 There was no difference in the FAAH expression at mRNA level between female IBS-D patients and healthy controls (Figure 3). However, a significant decrease in FAAH mRNA levels was detected in colonic biopsies from female IBS-C patients compared to healthy controls.

## Discussion

 In this preliminary study on a small cohort of IBS patients we have shown that the biolipid turnover may play a crucial role in the pathophysiology of IBS and the levels of FAAH metabolites correlate with the motility pattern and disease symptoms. We have also observed that FAAH mRNA levels are correlated with the IBS type and a significant decrease in FAAH mRNA expression was confirmed in patients suffering from IBS-C. Finally, we have demonstrated that the biolipid turnover in IBS, although greatly dependent on FAAH activity, is also regulated by other enzymatic pathways, which need further elucidation.

Cannabinoids have been used for centuries for recreational purposes, but only relatively recently their potential clinical action attracted much attention. Although the localization and function of the components of ECS are not fully understood and are still under investigation in several systems [18,19], it has become evident that they play a crucial role in functional GI disorders, such as IBS, and targeting the ECS may become one of major sites of action of anti-IBS treatments. Targeting CB receptors, which are localized on the neural, immune and other non-neural cells, by endogenous and exogenous cannabinoids may alter central or enteric GI muscle excitation [20] and visceral hypersensitivity (for review, see: [6]). However, in extensive clinical studies CB receptor ligands, like dronabinol and Δ9-tetrahydrocannabinol, failed to produce a simultaneous relief of both major symptoms of IBS, pain and changes in motility patterns [21]; they were also shown to act in the central nervous system and thus produced side effects at higher doses, which may have masked the potential beneficial action [22]. In contrast, FAAH and MAGL blockers produce potent antinociceptive effects and regulate GI motility. However, behavioral side effects typical for CB agonists are absent only in animals treated with selective FAAH [23,24], but not selective MAGL or mixed FAAH/MAGL inhibitors [25–27]. Moreover, sustained FAAH blockade does not alter CB1 receptor function and lacks dependence liability [28,29], suggesting that blocking the enzyme is a promising anti-IBS strategy. FAAH as a potential target in IBS treatment has also been proposed by the group of M. Camilleri, who investigated genetic variation in endocannabinoid metabolism in IBS patients [21,30,31].

Our study suggests that the motility patterns in IBS patients may correlate with biolipid serum levels. The endocannabinoid and cannabinoid-like fatty acid amide homeostasis is finely tuned in human body by synthesis on demand and rapid intracellular degradation, in which both degrading enzymes and transmembrane transporters are involved. Our observations require thus further extension, since prolonged changes in the biolipid equilibrium may have both, diagnostic and therapeutic implications. Furthermore, our data suggests that not only typical endocannabinoids, but also biolipids binding to “non-classical” CB receptors may be involved in the IBS pathophysiology, what could be important for the understanding of the disease. Earlier studies focused mainly on AEA and 2-AG in the colon and small intestine and showed that AEA may be the major endocannabinoid influencing GI function [32,33]. However, it was later reported that cannabinoid-like fatty acid amides, such as PEA and OEA, can also be found in the GI tract and are able to reduce intestinal motility [34–36]. Although there is conflicting data on the involvement of the “non-classical” CB receptors in the anti-motility effect and the modulation of basal visceral sensitivity by PEA and OEA, which still needs to be elucidated [37–39], it is apparent that several components of the ECS play important roles in the development of IBS.

Finally, we have shown that the FAAH mRNA expression is decreased in patients with IBS-C. This suggests slower turnover of endocannabinoids and may in part elegantly explain the slowing of motility, a typical feature of IBS-C. However, lower FAAH mRNA levels are in contrast with decreased PEA concentrations found in the IBS-C patient plasma, since this fatty acid amide is one of the substrates for FAAH and should be accumulated in the absence of enzyme activity. This suggests that other degradation enzymes are involved in PEA metabolism in the course of IBS. On the other hand, since samples for quantification of FAAH mRNA expression and endocannabinoid levels originated from two separate groups of patients, this hypothesis needs further elucidation.

In conclusion, our study shows that two major pillars of the ECS, the enzyme FAAH and its metabolites, correlate with IBS subtypes and IBS symptoms, whereas the basal release of the biolipids is not altered. Here we report that the alterations in endocannabinoids or cannabinoid-like fatty acid amides are associated with symptoms arising from altered motility and nociception and suggest their pathophysiological involvement. For IBS-C, the increased OEA levels may be due to the reduced FAAH expression and in consequence contribute to delayed intestinal transit in these patients. In contrast, the reduced OEA levels in patients with IBS-D may contribute to the increased GI transit. Our study furthermore provides evidence that the biolipid PEA level is lower in patients who report daily cramping and we suggest that the lack of PEA, which was shown to induce antinociception in animals, is an important component underlying the increased abdominal pain in patients with both IBS subtypes. Finally, our study for the first time shows that altered FAAH expression and distinct alterations in ECB or cannabinoid-like fatty acid amide levels are associated with motility phenomena like diarrhea and constipation, as well as symptoms of increased nociception and constitute that the endocannabinoid system is a crucial player in the pathophysiology of IBS. However, since samples for quantification of FAAH mRNA expression and endocannabinoid levels originated from two separate groups of patients, this hypothesis needs further elucidation.

## References

[1] Jones R, Lydeard S (1992) Irritable bowel syndrome in the general population. BMJ 304: 87-90. doi:10.1136/bmj.304.6819.87. PubMed: 1737146. 1737146 PMC1880997

[2] Talley NJ, Zinsmeister AR, Van DC, Melton LJ III (1991) Epidemiology of colonic symptoms and the irritable bowel syndrome. Gastroenterology 101: 927-934. PubMed: 1889716. 1889716 10.1016/0016-5085(91)90717-y

[3] Jones J, Boorman J, Cann P, Forbes A, Gomborone J et al. (2000) British Society of Gastroenterology guidelines for the management of the irritable bowel syndrome. Gut 47 Suppl 2: ii1-i19. doi:10.1136/gut.47.1.1. PubMed: 11053260. 11053260 PMC1766762

[4] Sandler RS, Drossman DA, Nathan HP, McKee DC (1984) Symptom complaints and health care seeking behavior in subjects with bowel dysfunction. Gastroenterology 87: 314-318. PubMed: 6735075. 6735075

[5] Akbar A, Walters JR, Ghosh S (2009) Review article: visceral hypersensitivity in irritable bowel syndrome: molecular mechanisms and therapeutic agents. Aliment Pharmacol Ther 30: 423-435. doi:10.1111/j.1365-2036.2009.04056.x. PubMed: 19493256. 19493256

[6] Storr MA, Sharkey KA (2007) The endocannabinoid system and gut-brain signalling. Curr Opin Pharmacol 7: 575-582. doi:10.1016/j.coph.2007.08.008. PubMed: 17904903. 17904903

[7] Kulkarni-Narla A, Brown DR (2000) Localization of CB1-cannabinoid receptor immunoreactivity in the porcine enteric nervous system. Cell Tissue Res 302: 73-80. doi:10.1007/s004410000261. PubMed: 11079717. 11079717

[8] Coutts AA, Irving AJ, Mackie K, Pertwee RG, Anavi-Goffer S (2002) Localisation of cannabinoid CB(1) receptor immunoreactivity in the guinea pig and rat myenteric plexus. J Comp Neurol 448: 410-422. doi:10.1002/cne.10270. PubMed: 12115703. 12115703

[9] Bridges D, Rice AS, Egertová M, Elphick MR, Winter J et al. (2003) Localisation of cannabinoid receptor 1 in rat dorsal root ganglion using in situ hybridisation and immunohistochemistry. Neuroscience 119: 803-812. doi:10.1016/S0306-4522(03)00200-8. PubMed: 12809701. 12809701

[10] Casu MA, Porcella A, Ruiu S, Saba P, Marchese G et al. (2003) Differential distribution of functional cannabinoid CB1 receptors in the mouse gastroenteric tract. Eur J Pharmacol 459: 97-105. doi:10.1016/S0014-2999(02)02830-3. PubMed: 12505538. 12505538

[11] MacNaughton WK, Van S, Keenan CM, Cushing K, Mackie K et al. (2004) Distribution and function of the cannabinoid-1 receptor in the modulation of ion transport in the guinea pig ileum: relationship to capsaicin-sensitive nerves. Am J Physiol Gastrointest Liver Physiol 286: G863-G871. doi:10.1152/ajpgi.00482.2003. PubMed: 14701723. 14701723

[12] Croxford JL (2003) Therapeutic potential of cannabinoids in CNS disease. CNS Drugs 17: 179-202. doi:10.2165/00023210-200317030-00004. PubMed: 12617697. 12617697

[13] Ueda N, Yamamoto S (2000) Anandamide amidohydrolase (fatty acid amide hydrolase). Prostaglandins Other Lipid Mediat 61: 19-28. doi:10.1016/S0090-6980(00)00052-6. PubMed: 10785539. 10785539

[14] Di M, V, De PL, Bisogno T (2001) Endocannabinoids Part I: molecular basis of endocannabinoid formation, action and inactivation and development of selective inhibitors. Expert Opin Ther Targets 5: 241-265. doi:10.1517/14728222.5.2.241. PubMed: 15992179. 15992179

[15] Folch J, Lees M, Sloane Stanley GH (1957) A simple method for the isolation and purification of total lipides from animal tissues. J Biol Chem 226: 497-509. PubMed: 13428781. 13428781

[16] Williams J, Wood J, Pandarinathan L, Karanian DA, Bahr BA et al. (2007) Quantitative method for the profiling of the endocannabinoid metabolome by LC-atmospheric pressure chemical ionization-MS. Anal Chem 79: 5582-5593. doi:10.1021/ac0624086. PubMed: 17600384. 17600384

[17] Wood JT, Williams JS, Pandarinathan L, Courville A, Keplinger MR, Janero et al. (2008) Comprehensive profiling of the human circulating endocannabinoid metabolome: clinical sampling and sample storage parameters. Clin Chem Lab Med 46: 1289-1295. PubMed: 18611105. 18611105 10.1515/CCLM.2008.242 PMC3733471

[18] Brocato B, Zoerner AA, Janjetovic Z, Skobowiat C, Gupta S, et al. (2013) Endocannabinoid crosstalk between placenta and maternal fat in a baboon model (Papio spp.) of obesity. Placenta 34: 983-989. S0143-4004(13)00692-9 [pii];Available online at: 10.1016/j.placenta.2013.08.007 [doi] 24008071 PMC3827983

[19] Slominski AT, Zmijewski MA, Skobowiat C, Zbytek B, Slominski RM, et al. (2012) Sensing the environment: regulation of local and global homeostasis by the skin's neuroendocrine system. Adv Anat Embryol Cell Biol 212: v, vii, 1-v, vii115 22894052 10.1007/978-3-642-19683-6_1 PMC3422784

[20] Esfandyari T, Camilleri M, Busciglio I, Burton D, Baxter K et al. (2007) Effects of a cannabinoid receptor agonist on colonic motor and sensory functions in humans: a randomized, placebo-controlled study. Am J Physiol Gastrointest Liver Physiol 293: G137-G145. doi:10.1152/ajpgi.00565.2006. PubMed: 17395895. 17395895

[21] Wong BS, Camilleri M, Busciglio I, Carlson P, Szarka LA et al. (2011) Pharmacogenetic trial of a cannabinoid agonist shows reduced fasting colonic motility in patients with nonconstipated irritable bowel syndrome. Gastroenterology 141: 1638-1647. doi:10.1053/j.gastro.2011.07.036. PubMed: 21803011. 21803011 PMC3202649

[22] Fox A, Bevan S (2005) Therapeutic potential of cannabinoid receptor agonists as analgesic agents. Expert Opin Investig Drugs 14: 695-703. doi:10.1517/13543784.14.6.695. PubMed: 16004597. 16004597

[23] Cravatt BF, Demarest K, Patricelli MP, Bracey MH, Giang DK et al. (2001) Supersensitivity to anandamide and enhanced endogenous cannabinoid signaling in mice lacking fatty acid amide hydrolase. Proc Natl Acad Sci U S A 98: 9371-9376. doi:10.1073/pnas.161191698. PubMed: 11470906. 11470906 PMC55427

[24] Kathuria S, Gaetani S, Fegley D, Valiño F, Duranti A et al. (2003) Modulation of anxiety through blockade of anandamide hydrolysis. Nat Med 9: 76-81. PubMed: 12461523. 12461523 10.1038/nm803

[25] Ramesh D, Ross GR, Schlosburg JE, Owens RA, Abdullah RA et al. (2011) Blockade of endocannabinoid hydrolytic enzymes attenuates precipitated opioid withdrawal symptoms in mice. J Pharmacol Exp Ther 339: 173-185. doi:10.1124/jpet.111.181370. PubMed: 21719468. 21719468 PMC3186294

[26] Long JZ, Nomura DK, Cravatt BF (2009) Characterization of monoacylglycerol lipase inhibition reveals differences in central and peripheral endocannabinoid metabolism. Chem. Biol 16: 744-753. 10.1016/j.chembiol.2009.05.009 PMC2867454 19635411

[27] Long JZ, Nomura DK, Vann RE, Walentiny DM, Booker L et al. (2009) Dual blockade of FAAH and MAGL identifies behavioral processes regulated by endocannabinoid crosstalk in vivo. Proc Natl Acad Sci U S A 106: 20270-20275. doi:10.1073/pnas.0909411106. PubMed: 19918051. 19918051 PMC2787168

[28] Schlosburg JE, Carlson BL, Ramesh D, Abdullah RA, Long JZ et al. (2009) Inhibitors of endocannabinoid-metabolizing enzymes reduce precipitated withdrawal responses in THC-dependent mice. AAPS J 11: 342-352. doi:10.1208/s12248-009-9110-7. PubMed: 19430909. 19430909 PMC2691470

[29] Schlosburg JE, Blankman JL, Long JZ, Nomura DK, Pan B et al. (2010) Chronic monoacylglycerol lipase blockade causes functional antagonism of the endocannabinoid system. Nat Neurosci 13: 1113-1119. doi:10.1038/nn.2616. PubMed: 20729846. 20729846 PMC2928870

[30] Wong BS, Camilleri M, Eckert D, Carlson P, Ryks M et al. (2012) Randomized pharmacodynamic and pharmacogenetic trial of dronabinol effects on colon transit in irritable bowel syndrome-diarrhea. Neurogastroenterol Motil 24: 358-e169. doi:10.1111/j.1365-2982.2011.01874.x. PubMed: 22288893. 22288893 PMC3775711

[31] Camilleri M, Carlson P, McKinzie S, Grudell A, Busciglio I, et al. (2008) Genetic variation in endocannabinoid metabolism, gastrointestinal motility, and sensation. Am J Physiol Gastrointest Liver Physiol 294: G13-G19 00371.2007 [pii];Available online at: 10.1152/ajpgi.00371.2007 [doi] 17962356

[32] Izzo AA, Fezza F, Capasso R, Bisogno T, Pinto L et al. (2001) Cannabinoid CB1-receptor mediated regulation of gastrointestinal motility in mice in a model of intestinal inflammation. Br J Pharmacol 134: 563-570. doi:10.1038/sj.bjp.0704293. PubMed: 11588110. 11588110 PMC1572987

[33] Obata T, Sakurai Y, Kase Y, Tanifuji Y, Horiguchi T (2003) Simultaneous determination of endocannabinoids (arachidonylethanolamide and 2-arachidonylglycerol) and isoprostane (8-epiprostaglandin F2alpha) by gas chromatography-mass spectrometry-selected ion monitoring for medical samples. J Chromatogr B Analyt Technol Biomed Life Sci 792: 131-140. doi:10.1016/S1570-0232(03)00311-8. PubMed: 12829006. 12829006

[34] Capasso R, Izzo AA, Fezza F, Pinto A, Capasso F et al. (2001) Inhibitory effect of palmitoylethanolamide on gastrointestinal motility in mice. Br J Pharmacol 134: 945-950. doi:10.1038/sj.bjp.0704339. PubMed: 11682441. 11682441 PMC1573032

[35] Pinto L, Izzo AA, Cascio MG, Bisogno T, Hospodar-Scott K et al. (2002) Endocannabinoids as physiological regulators of colonic propulsion in mice. Gastroenterology 123: 227-234. doi:10.1053/gast.2002.34242. PubMed: 12105851. 12105851

[36] Cluny NL, Keenan CM, Lutz B, Piomelli D, Sharkey KA (2009) The identification of peroxisome proliferator-activated receptor alpha-independent effects of oleoylethanolamide on intestinal transit in mice. Neurogastroenterol Motil 21: 420-429. doi:10.1111/j.1365-2982.2008.01248.x. PubMed: 19140957. 19140957

[37] Brusberg M, Arvidsson S, Kang D, Larsson H, Lindström E et al. (2009) CB1 receptors mediate the analgesic effects of cannabinoids on colorectal distension-induced visceral pain in rodents. J Neurosci 29: 1554-1564. doi:10.1523/JNEUROSCI.5166-08.2009. PubMed: 19193902. 19193902 PMC6666086

[38] Lambert DM, Vandevoorde S, Jonsson KO, Fowler CJ (2002) The palmitoylethanolamide family: a new class of anti-inflammatory agents? Curr Med Chem 9: 663-674. doi:10.2174/0929867023370707. PubMed: 11945130. 11945130

[39] Li K, Fichna J, Schicho R, Saur D, Bashashati M et al. (2013) A role for O-1602 and G protein-coupled receptor GPR55 in the control of colonic motility in mice. Neuropharmacology 71: 255-263. doi:10.1016/j.neuropharm.2013.03.029. PubMed: 23603203. 23603203 PMC3677091

